# Spontaneous Massive Hemorrhage Caused by Inflammatory Myofibroblastic Tumor (IMT) of the Greater Omentum: A Case Report

**DOI:** 10.7759/cureus.103499

**Published:** 2026-02-12

**Authors:** Wanbin He, Jie Dan, Mingjie Zhu, Chengjun Zheng, Yonghong Wang

**Affiliations:** 1 Department of Gastrointestinal Surgery, The People's Hospital of Leshan, Leshan, CHN

**Keywords:** case report, greater omentum, inflammatory myofibroblastic tumor (imt), metastases, spontaneous massive hemorrhage

## Abstract

Inflammatory myofibroblastic tumor (IMT) is a rare mesenchymal soft tissue neoplasm. Its histologic features include proliferating fibroblastic/myofibroblastic spindle cells and a dense inflammatory infiltrate rich in plasma cells, lymphocytes, and eosinophils. However, it can exhibit aggressive behavior, including local invasion, malignant transformation, recurrence, and even metastasis.

We report a case of a 27-year-old man who experienced a spontaneous massive hemorrhage caused by IMT of the greater omentum and who had previously undergone right upper lobectomy for the IMT of the lung. Furthermore, we deduced that there were metastatic tumors in the sternum, thoracic vertebrae, lumbar vertebrae, ilium, sacrum, and right femur, as well as in soft tissue masses located in the retroperitoneum and the right buttocks.

It presents with diverse clinical manifestations and lacks specific imaging characteristics. Definitive diagnosis relies on histopathological examination and immunohistochemical analysis. Given that these tumors may recur locally and, in rare cases, undergo malignant transformation, targeted therapy may be warranted. Timely diagnosis and surgical resection are crucial, and long-term follow-up is essential for ongoing surveillance.

## Introduction

The inflammatory myofibroblastic tumor (IMT) is a rare mesenchymal neoplasm characterized by the proliferation of myofibroblasts accompanied by a prominent inflammatory cell infiltrate. Although typically considered to have low malignant potential, IMT can exhibit aggressive behavior, including local invasion, recurrence, and even metastasis [1]. Its exact etiology and pathogenesis remain unclear. IMT is primarily a disease of children and young adults; sporadic cases occur across the entire age spectrum [2]. These tumors can arise in a wide range of sites, affecting virtually any organ system. The disease shows a predilection for the lung, while other sites, in descending order of frequency, are soft tissues, bone, the abdominopelvic cavity, the head and neck region, and the retroperitoneum, with esophageal, cardiac, and adrenal involvement being comparatively uncommon [3]. Positive diagnosis relies on clinical and radiological findings combined with preoperative cytohistological examination. However, as these methods are not always definitive, surgical resection is often performed for both definitive diagnosis and therapy, particularly given the tumor's potential for invasiveness and postoperative recurrence [4]. Due to its rarity and complex clinicopathological features, IMT remains a diagnostic challenge, necessitating a coordinated multidisciplinary review of radiological and histopathological data. We report a challenging case of spontaneous, life-threatening hemorrhage arising from an IMT of the greater omentum, a manifestation exceedingly rare in the literature, which renders this case uniquely instructive for clinical practice. Adding further complexity, the patient had a prior history of surgery for pulmonary IMT-related hematemesis and was concurrently experiencing lumbar pain attributed to spinal metastasis. This case report is structured in accordance with the Surgical CAse REport (SCARE) criteria [5].

## Case presentation

History of surgery for pulmonary diseases

A 27-year-old man was admitted for a four-month history of blood-streaked sputum that progressed to frank hemoptysis on December 20, 2023 (Table 1), accompanied by a two-kg weight loss. The patient denied coughing, night sweats, chest pain, dyspnea, or a family history of cancer, and he was not taking any medication at home. The result of hemoglobin (Hb) was severe anemia at 42 g/L (reference range: 115-150 g/L), and a heart rate (HR) of 121 beats per minute with a blood pressure (BP) of 118/65 mmHg, which prompted blood transfusion immediately.

**Table 1:** Patient timeline CT: computed tomography, MDT: multidisciplinary team, ED: emergency department, IMT: inflammatory myofibroblastic tumor

Time (Date)	Event	Findings/Diagnosis	Management
Dec 20, 2023	Admission for hemoptysis	Severe anemia (Hb 42 g/L)	Immediate transfusion
Dec 22, 2023	Chest CT imaging	8.1 cm mass, right upper lobe	-
Dec 25, 2023	Lung biopsy	Mesenchymal tumor (undefined subtype)	-
Jan 15, 2024	MDT	Surgical resection advised	Surgery
Jan 16, 2024	Right upper lobectomy	Pathology inconclusive post-op	Expert pathology consult (patient deferred)
Feb – Jun 2024	Asymptomatic interval	No examinations recorded	No active treatment
Jul 19, 2024	ED visit (acute abdomen)	Omental mass, multiple metastases identified	Non-operative management
Jul 22, 2024	Clinical deterioration	Hemoperitoneum (Hb 79 g/L)	Emergency laparotomy + transfusion
Sep 30, 2024	External pathology review	Final diagnosis: ALK-positive IMT	Diagnosis confirmed
Oct 2024	Systemic therapy starts	ALK rearrangement confirmed	Oral ensartinib initiated
Oct 2025	Follow-up contact	Stable symptoms, no imaging surveillance	Ongoing targeted therapy

**Figure 1:**
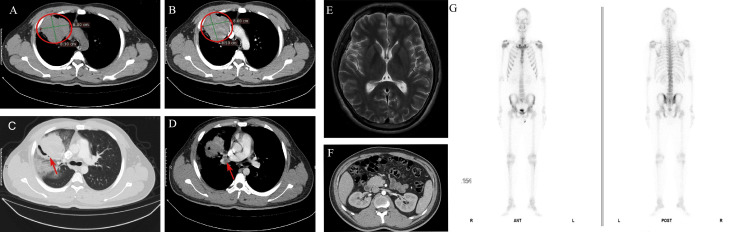
Imaging findings Chest CT scan (A, red circle, mediastinal window) and enhanced CT (B, red circle, mediastinal window) in axial planes demonstrate a large soft tissue mass measuring approximately 8.1 cm × 8.0 cm in the right upper lobe, accompanied by alveolar hemorrhage, partial bronchial obstruction (C, red arrow, lung window), and enlargement of right hilar lymph nodes (D, red arrow, mediastinal window). No lesions were identified in the brain (E, MRI), abdominal organs (F, contrast-enhanced CT), or bones (G, emission computed tomography (ECT)).

Computed tomography (CT) of the chest performed on December 22, 2023, showed a large soft tissue mass measuring 8.1 cm × 8 cm in the right upper lobe, partially obstructing the bronchus, with alveolar hemorrhage and enlargement of right hilar lymph nodes (Figure 1). No secondary lesions were detected in the brain, abdominal organs, or skeletal system. The emission CT (ECT) revealed no abnormalities, with findings reported as negative for metastatic disease.

**Figure 2:**
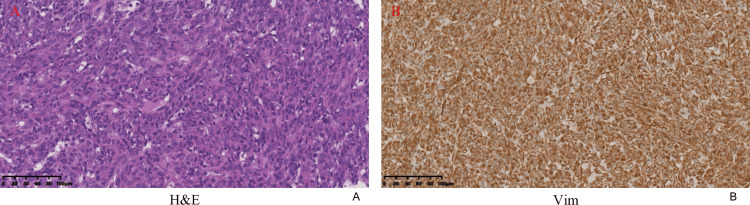
Needle biopsy findings The needle biopsy pathology of the lung mass revealed a mesenchymal tumor (A), and the immunohistochemical evaluation showed Vim (+) (B).

The December 25, 2023, needle biopsy of the lung mass revealed a mesenchymal tumor and the immunohistochemical evaluation showed: CK (-), EMA (-), BcI-2 (-), Vim (+), CK7 (-), TTF-1 (-), P40 (-), Syn (-), S-100 (-), STAT-6 (-), CD34 (+), CD99 (-), Ki67 (+, 5%), WT-1 (-), SSTR2 (-), HMB-45 (-), MeIan-A (-), TLE1 (-), Desmin (-), SMA (-), CD20 (-), P63(-) (Figure 2).

A multidisciplinary team (MDT) discussion was held on January 15, 2024, by the departments of thoracic surgery, imaging, pathology, and oncology. Based on the expert opinion of the MDT, the patient who suffered from persistent hemoptysis was diagnosed with a large tumor in the right upper lobe, and the specific type of tumor remains undetermined by pathology. Therefore, the patient was advised to receive surgery.

**Figure 3:**
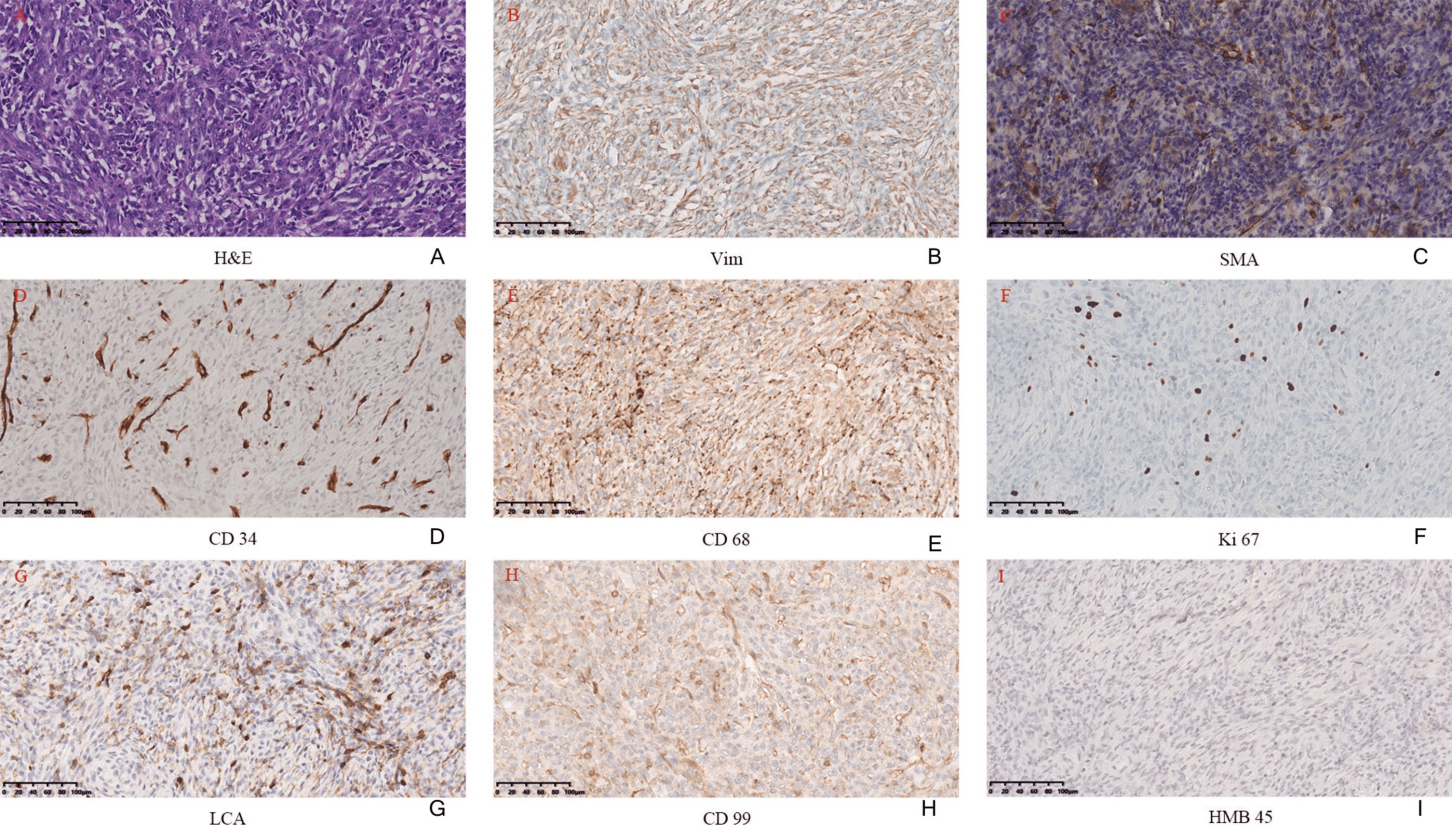
Microscopic view findings The microscopic view showed two components: blood vessels and spindle cells (A). Immunohistochemical stains showed that Vim (B), SMA (C), CD34 (D), CD68 (E), Ki67 (F), LCA (G), and CD99 (H) were positive, and HMB-45 (I) was negative.

On January 16, 2024, a right upper lobectomy was performed after a blood transfusion to correct anemia with 8.5 units of suspended red blood cells. The operation and the postoperative course were uneventful, and the patient was discharged from the hospital four days after surgery. The postoperative immunohistochemical evaluation showed: Vim (+), P-CK (-), HMB-45 (-), MeIan-A (-), SMA (+), Desmin (-), S-100 (-), CD34 (+), CD68 (+), Ki67 (+, 30%), CD117 (-), CK19 (-), EMA (-), TLE-1 (-), Actin (-), MyoD1(-), LCA (+), WT-1 (-), Myogenin (-), CR (-), CD1α (-), CD99 (+), CD23 (-), CD35, and Langerin (-) (Figure 3).

Complete resection was confirmed by histopathology without lymph node metastasis. However, given the inconclusive tumor typing results at our institution, referral to a higher-level hospital for expert consultation was recommended. The patient reported no discomfort after discharge and declined pathological consultation due to personal reasons. Between February and June 2024, no examinations or treatments were performed.

Spontaneous massive hemorrhage of an omental tumor

**Figure 4:**
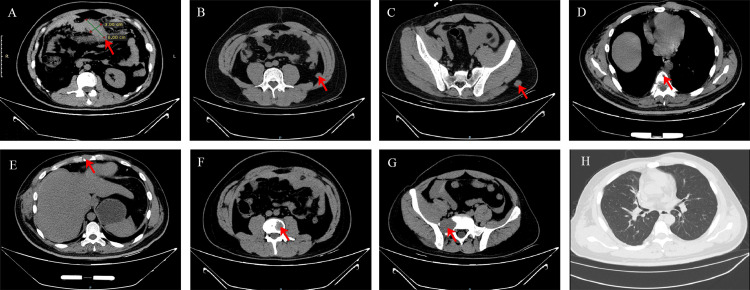
Non-contrast CT of the abdomen Non-contrast CT of the abdomen in axial planes reveals a soft tissue mass measuring approximately 6 cm × 3 cm in the greater omentum region (A, red arrow). Additional soft tissue masses are identified in the retroperitoneum (B, red arrow) and the left buttock (C, red arrow). New multiple osteolytic bone destructions are noted in the thoracic vertebrae (D, red arrow), sternum (E, red arrow), lumbar vertebrae (F, red arrow), and sacrum (G, red arrow). No significant lesions are observed in the lungs (H).

On July 19, 2024, the patient was evaluated in the emergency department for severe abdominal pain. Additionally, he had been complaining of a backache for a few months. The emergency CT of the abdomen showed a mass approx. 6 cm × 3 cm in the omental region with a small amount of hemoperitoneum. Multiple new osteolytic lesions were identified in the sternum, thoracic vertebrae, lumbar vertebrae, and sacrum, along with soft tissue masses in the retroperitoneum and left buttock (Figure 4), indicating disease progression during this interval. No lesions were observed in the lung. The level of Hb was at 141 g/L. Thus, the patient was treated with non-operative treatment.

**Figure 5:**
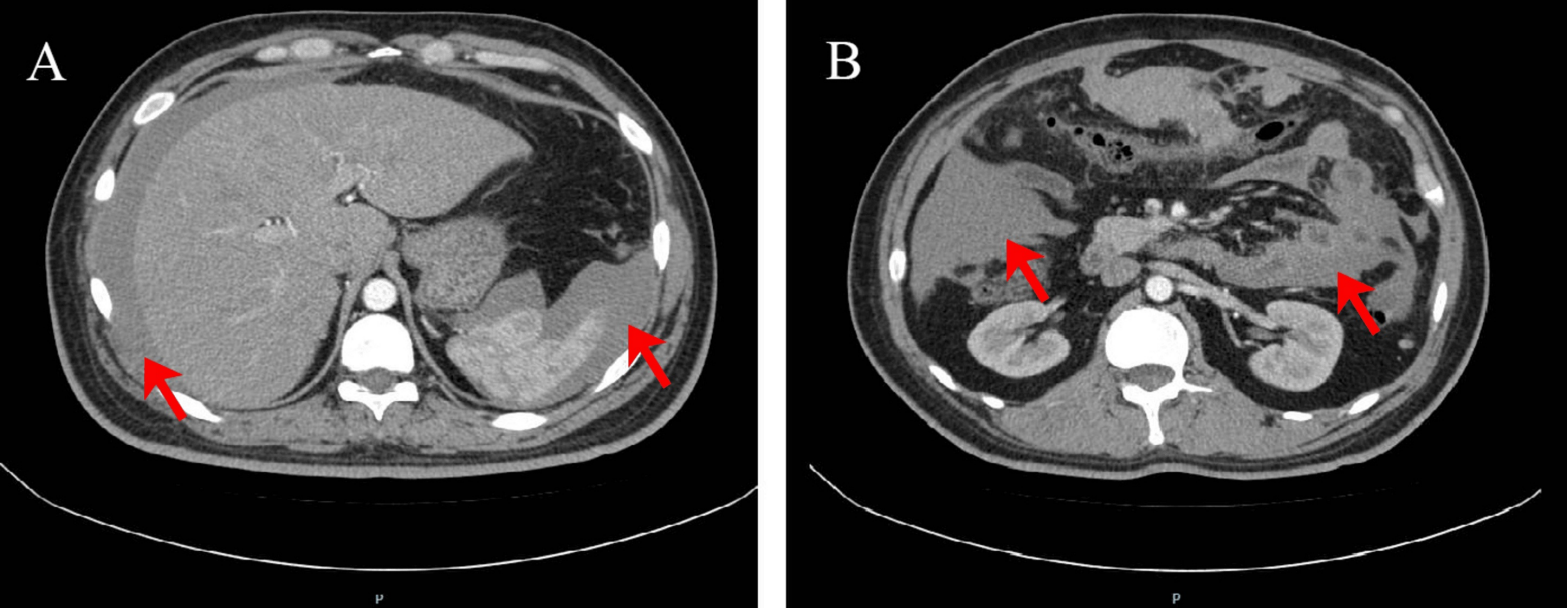
Contrast-enhanced abdominal CT Contrast-enhanced abdominal CT in axial planes, compared to the prior examination, reveals an increase in hemoperitoneum surrounding the liver and spleen (A, red arrow), along with an increased accumulation of blood within the interintestinal spaces (B, red arrow).

**Figure 6:**
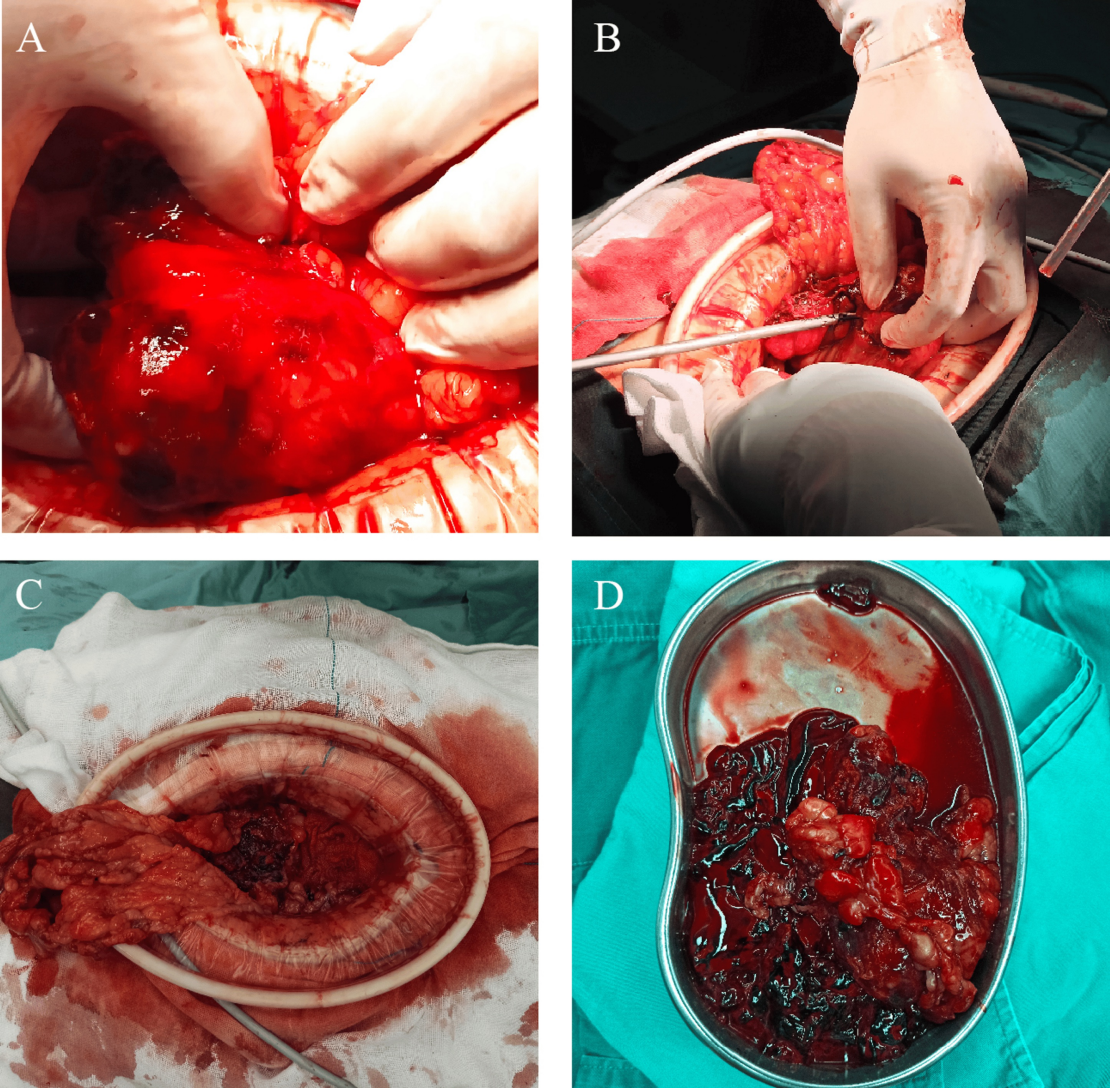
Intraoperative findings Revealed a tumor measuring 3.5 cm × 3 cm between the omental region and transverse colon (A, D). Resection of the tumor using an ultrasonic scalpel (B). No bleeding was observed at the surgical wound after the resection of the tumor (C).

**Figure 7:**
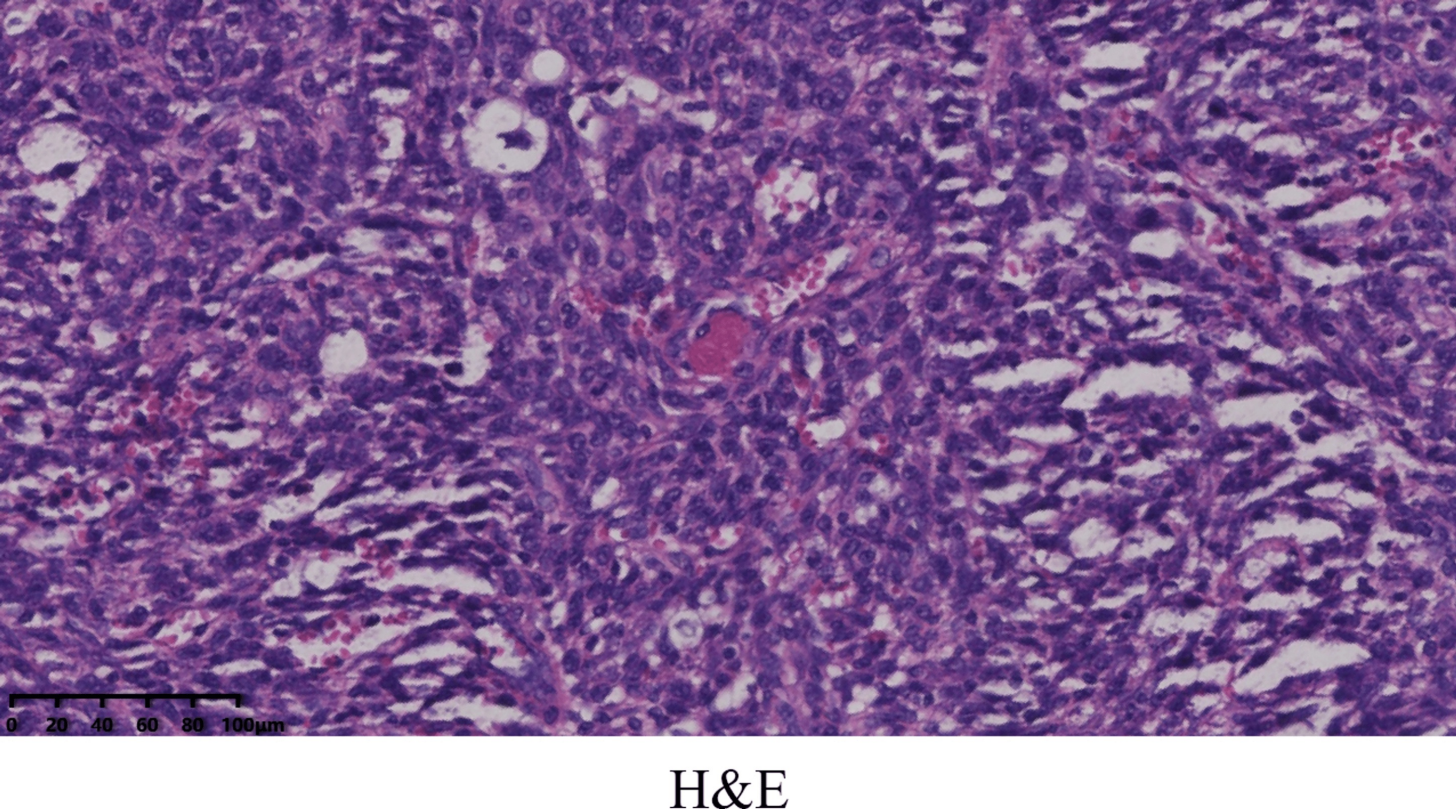
The tumor involved numerous spindle-shaped cells

However, the abdominal pain of the patient intensified sharply on July 22, 2024. An enhanced CT scan demonstrated a hemoperitoneum larger than before (Figure 5), and the level of Hb was at 79 g/L. As the patient remained hemodynamically unstable, the patient underwent an exploratory laparotomy, during which he received a transfusion of four units of red blood cells. Intraoperative findings revealed a tumor measuring 3.5 cm × 3 cm between the omental region and transverse colon (Figure 6), which was actively bleeding and surrounded by a significant blood clot, and 1.4 liters of blood had been collected from the peritoneal cavity. Histopathologically, the tumor involved numerous spindle-shaped cells (Figure 7).

**Figure 8:**
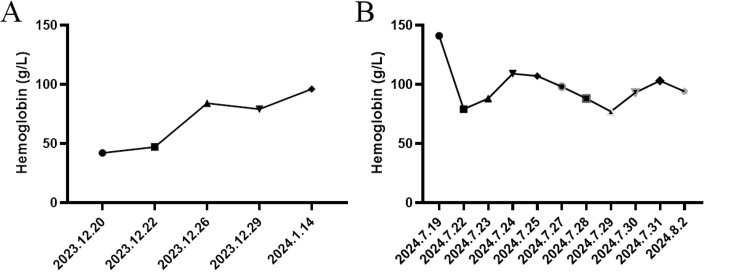
Changes in hemoglobin (Hb) levels During lung surgery (A) and abdominal surgery (B).

The patient was moved to the ICU for two days, where the patient was extubated and received transfusion of five units of red blood cells and 450 ml plasma, then transferred to the normal ward uneventfully. Thereafter, approximately 350 ml hemorrhagic fluid was collected from the peritoneal drain tube, followed by a transfusion of four units of red blood cells for the patient, and the patient was discharged from the hospital 16 days after surgery (Figure 8).

Pathological consultation and final diagnosis

On September 30, 2024, a pathological consultation for tumors was obtained at a tertiary hospital; the immunohistochemical evaluation showed that Desmin (-), SMA (-), CD34 (vessel +), CD31 (vessel +), ALK (+), HMB-45(-), MITF (-), STAT6 (-), S-100 (-), SOX10 (-), H3K27ME3 (retained), MART-1 (-), PCK (-), EMA (-), Brachyury (-), INI-1 (retained), TRK-Pan (-), CD68 (PGM-1) positive in histiocytes, TLE-1 (-), SALL4(-), Ki67 (+, 15%), CD163 (positive in partial histiocytes), PAS (-), PAS with digestion (-), AB (-) (Table 2). Fluorescence in situ hybridization (FISH) test: An unbalanced translocation of the ALK gene was detected; no significant ESR1 gene translocation was identified.

**Table 2:** Immunohistochemistry (IHC) results

Time	Positive Markers	Negative Markers
December 25, 2023	CD34, Ki67 (5%) CD99, Vim	HMB-45, MeIan-A, S-100, TLE1 BcI-2, CD20, CK, CK7, Desmin, EMA, P40, P63, SMA, SSTR2, STAT-6, Syn, TTF-1, WT-1
January 16, 2024	CD34, Ki67 (30%) CD68, CD99, LCA, SMA, Vim	HMB-45, MeIan-A, S-100, TLE-1 Actin, CD1α, CD117, CD23, CD35, CK19, CR, Desmin, EMA, Langerin, MyoD1, Myogenin, P-CK, WT-1
September 30, 2024 (Pathology Consultation)	CD34 (vessel), Ki67 (15%) ALK, CD31 (vessel), CD68 (PGM-1 histiocytes), INI-1, CD163 (partial histiocytes)	HMB-45, MART-1, S-100, TLE-1, AB, Brachyury, Desmin, EMA, H3K27ME3, MITF, PAS, PCK, SALL4, SMA, SOX10, STAT6, TRK-Pan

Comprehensive diagnosis

The comprehensive diagnosis was in favor of IMT (borderline tumor). Given increased cellular density and focal epithelioid morphology, further next-generation sequencing (NGS) testing is recommended. However, the patient did not undergo further examination.

Treatment and follow-up

In October 2024, a higher-level hospital recommended oral targeted therapy. Due to financial considerations, the patient opted for the more affordable ensartinib, initiating treatment at a dose of 225 mg once daily orally. This treatment is to be continued until disease progression or unacceptable toxicity. During a telephone follow-up in October 2025, the patient reported ongoing oral intake of ensartinib, describing their general condition as fair but noting intermittent back pain. Since discharge, the patient has not undergone any imaging studies at any medical facility.

## Discussion

IMT is quite rare, and its exact epidemiological data are still unclear. Since its annual incidence is less than 1/1000000, which was defined as one of the “ultra- rare sarcomas” at the 2019 Connective Tissue Oncology Society Consensus [6]. Within the 2020 WHO schema, IMTs occupy a category of low-grade malignancy, characterized as borderline tumors that may metastasize or recur [7]. A subset of IMTs, often those located in the abdominal cavity, demonstrate malignant potential or low-grade malignancy with a poorer prognosis, despite most tumors exhibiting an indolent behavior [8]. IMT most commonly manifests in the lung, where it represents only 0.04%-1% of all lung tumors and is more often parenchymal than endobronchial [4]. The diagnosis of IMT is challenging due to its highly variable and nonspecific clinical presentation, which encompasses symptoms, systemic manifestations, and imaging findings that overlap with many other tumors [9]. The clinical manifestations of IMT are largely determined by the anatomic site of the primary lesion. In the present case, the patient presented with hemoptysis and abdominal pain during two distinct hospital admissions. This highlights the absence of pathognomonic symptoms in IMT and its frequent incidental diagnosis. The patient's reported back pain could potentially be attributed to vertebral involvement. Notably, massive intra-abdominal hemorrhage resulting from omental seeding of IMT has not been previously reported in the literature.

The imaging characteristics of IMT are notably heterogeneous, which stems from variations in their fibrous and cellular content [10]. CT and MRI constitute the fundamental diagnostic modalities; however, specific imaging characteristics for IMT are lacking, and radiological findings typically reveal solid, regular, well-defined masses, contingent upon the primary tumor’s location [11]. IMT typically appears on CT as homogeneous or heterogeneous lesions with mild to moderate enhancement, ranging from infiltrative to well-delineated masses containing inflammatory and fibrotic components that may lead to delayed uptake; calcification is generally absent, and its presence suggests alternative malignancies [3]. Typically located in the lower lobes, pulmonary IMTs are often peripheral, well-defined, and exhibit a lobulated morphology on imaging [12]. Abdominal IMTs typically appear as round, oval, or irregular low-density lesions; depending on their composition, contrast enhancement can be heterogeneous or homogeneous, peripheral, or progressive [13]. For evaluating the extent and soft tissue involvement of IMT, MRI is the preferred modality over CT. On MRI, IMT typically shows hypointense signal in both T1 and T2 sequences but can exhibit T2 hyperintensity with marked enhancement, suggesting rapid growth and aggressiveness, while peritumoral edema often indicates a more benign course and helps define surgical margins [14]. Despite its limited utility in primary diagnosis due to the mixed histology of IMT, PET-CT remains valuable for detecting primary tumors, assessing recurrence and metastasis, and monitoring treatment response [15]. Therefore, while imaging can raise suspicion for malignancy, IMT cannot be definitively diagnosed based on imaging alone.

Ultimately, a definitive diagnosis of IMT is established through histopathological examination and immunohistochemical staining. The histologic diagnosis of IMT rests on three key findings: a proliferation of spindle-shaped myofibroblasts; a polymorphous inflammatory infiltrate (typically of plasma cells, lymphocytes, eosinophils, and neutrophils); and variable stromal alterations such as fibrosis, hyalinization, calcification, or necrosis [16]. The pathological histology manifests in three patterns: nodular fasciitis-like (myxoid type): spindle-shaped fibroblasts/myofibroblasts proliferate within a myxoid edematous intercellular content; hypercellular type: spindle-shaped fibroblasts/myofibroblasts are densely arranged in bundles; hypocellular type: hyaline collagenous intercellular content with sparse spindle cells [17]. One tumor may predominantly show one histological pattern or contain multiple patterns. However, morphological manifestations are sometimes still insufficient to provide adequate diagnostic evidence, and immunohistochemistry (IHC) staining is essential for diagnosing IMT, as it aids in defining the immunophenotypic profile of myofibroblasts and ruling out alternative conditions. The immunostaining for ALK, positive in about 50-60% of cases, can present as cytoplasmic, membranous, perinuclear, or a dot-like pattern, and its variability is a direct reflection of the underlying ALK gene fusion partner [2]. Patients with ALK-positive IMTs generally have a more favorable prognosis, in contrast to those with ALK-negative tumors, which often display aggressive behavior and a greater tendency to metastasize [18]. Also, ALK-positive tumors are found mostly in younger patients, with a predilection for patients under 40 years of age [3]. Nevertheless, for institutions lacking experience in diagnosing IMT, the identification of this disease indeed presents difficulties and may even lead to the inability to establish a definitive diagnosis or result in misdiagnosis. Given that the tumor typing for this patient was inconclusive at our hospital, a referral to a higher‑level medical institution for expert pathological consultation was recommended. However, as the patient reported no symptoms postoperatively, they declined the consultation and did not undergo close follow‑up between February and July 2024. It was precisely during this period that the disease progressed. Consequently, the definitive diagnostic report was not obtained from the higher‑level hospital until September 2024, and target therapy was subsequently started in October 2024. This course of events further highlights the diagnostic challenges posed by this type of disease at the primary‑care level.

Due to the rarity of the disease and the absence of large cohort analyses, current knowledge on the surgical management of IMT predominantly stems from case reports and case series, with total surgical excision or wide local excision remaining first‑line treatments, provided a complete resection encompassing the tumor and a margin of surrounding healthy tissue can achieve radical (R0) excision depending on tumor size and anatomical location [3]. Complete resection of IMT is associated with a favorable prognosis, demonstrating a five-year survival rate of 91% [19]. Some patients experience tumor recurrence after surgery. The recurrence rate of IMT varies by location after complete resection, with a 2% recurrence rate for pulmonary lesions and up to 25% for extrapulmonary lesions [17]. The patient's early CT/ECT examination revealed no evidence of multiple bone destruction, abdominal, or retroperitoneal metastasis. However, CT scans six months after R0 resection of the pulmonary lesion showed numerous new extrapulmonary lesions, suggesting that the disease may have entered a phase of rapid progression. This accelerated progression may have been related to the inability to confirm a pathological diagnosis at the time, which consequently delayed the initiation of targeted therapy. This case also highlights the risk of micrometastasis and supports considering adjuvant systemic therapy for aggressively behaving tumors. Consequently, long-term follow-up is mandatory for all IMT patients after excision. However, it should be noted that prospective studies on adjuvant therapy following complete resection are currently lacking. The National Comprehensive Cancer Network (NCCN) guidelines suggest using ALK inhibitors as the initial systemic treatment for advanced, recurrent, metastatic, or unresectable IMTs harboring ALK fusions [2]. A 2010 study demonstrated clinical responses to crizotinib in ALK-rearranged IMT patients, and recent case reports have further documented the efficacy of ensartinib in treating this condition [20]. Although targeted therapies for non-ALK fusion genes in IMT have been documented in case reports, robust clinical data are still lacking and necessitate further systematic research.

## Conclusions

IMTs represent a rare category of neoplasms exhibiting intermediate malignant potential. Pulmonary IMTs are both uncommon and diagnostically challenging, whereas spontaneous massive hemorrhage resulting from greater omental IMT is exceptionally rare. Diagnosing IMTs is difficult due to their resemblance to more aggressive malignancies. A definitive diagnosis frequently necessitates surgical excision, given the limitations of imaging and biopsy. These tumors may recur locally and, in rare instances, undergo malignant transformation. The latter possibility may necessitate targeted therapy. Therefore, long-term follow-up is essential for ongoing surveillance.

## References

[1] Khatri A, Agrawal A, Sikachi RR, Mehta D, Sahni S, Meena N (2018). Inflammatory myofibroblastic tumor of the lung. Adv Respir Med.

[2] Choi JH (2025). Inflammatory myofibroblastic tumor: sn updated review. Cancers (Basel).

[3] Chmiel P, SłOW A, Banaszek Ł (2024). Inflammatory myofibroblastic tumor from molecular diagnostics to current treatment. Oncol Res.

[4] Rhazari M, Thouil A, Gartini S, Kouismi H, Lakhal M (2025). Pulmonary inflammatory myofibroblastic tumor in a patient treated for pemphigus vulgaris: a case report. Cureus.

[5] (2025). Revised Surgical CAse REport (SCARE) Guideline: An update for the age of artificial intelligence. https://doi.org/10.70389/PJS.100079.

[6] Stacchiotti S, Frezza AM, Blay JY (2021). Ultra-rare sarcomas: a consensus paper from the Connective Tissue Oncology Society community of experts on the incidence threshold and the list of entities. Cancer.

[7] Choi JH, Ro JY (2021). The 2020 WHO classification of tumors of soft tissue: selected changes and new entities. Adv Anat Pathol.

[8] Fu GX, Xu CC, Yao NF, Gu JZ, Jiang HL, Han XF (2019). Inflammatory myofibroblastic tumor: a demographic, clinical and therapeutic study of 92 cases. Math Biosci Eng.

[9] Zhang Q, Zhang ZW, Fan J, Ji ZM, Wang CY, Liu F (2025). Clinical diagnosis and treatment of abdominal inflammatory myofibroblastic tumors. Discov Oncol.

[10] Sargar KM, Sheybani EF, Shenoy A, Aranake-Chrisinger J, Khanna G (2016). Pediatric fibroblastic and myofibroblastic tumors: a pictorial review. Radiographics.

[11] Surabhi VR, Chua S, Patel RP, Takahashi N, Lalwani N, Prasad SR (2016). Inflammatory myofibroblastic tumors: current update. Radiol Clin North Am.

[12] Lichtenberger JP 3rd, Biko DM, Carter BW, Pavio MA, Huppmann AR, Chung EM (2018). Primary lung tumors in children: radiologic-pathologic correlation from the radiologic pathology archives. Radiographics.

[13] Yamamura K, Beppu T, Oda E (2021). Hepatic inflammatory pseudotumor mimicking malignant tumor with rare onset of Intra-abdominal hemorrhage. Anticancer Res.

[14] Zeng X, Huang H, Li J, Peng J, Zhang J (2018). The clinical and radiological characteristics of inflammatory myofibroblastic tumor occurring at unusual sites. Biomed Res Int.

[15] Parker NC, Singanallur P, Faiek S, Gao J, White P (2024). Inflammatory myofibroblastic tumor after receiving treatment for non-small cell carcinoma. Cureus.

[16] Gros L, Dei Tos AP, Jones RL, Digklia A (2022). Inflammatory myofibroblastic tumour: state of the art. Cancers (Basel).

[17] Si X, Wu S, Feng R (2025). Chinese expert consensus on the diagnosis and treatment of inflammatory myofibroblastic tumor. Thorac Cancer.

[18] Arshad H, Crawford CK, Fishman EK (2025). Insights into inflammatory myofibroblastic tumor: a complex and challenging diagnosis. Radiol Case Rep.

[19] Sagar AE, Jimenez CA, Shannon VR (2018). Clinical and histopathologic correlates and management strategies for inflammatory myofibroblastic tumor of the lung. A case series and review of the literature. Med Oncol.

[20] Butrynski JE, D'Adamo DR, Hornick JL (2010). Crizotinib in ALK-rearranged inflammatory myofibroblastic tumor. N Engl J Med.

